# Superficial venous insufficiency from the infernal to the endothermal

**DOI:** 10.1308/003588414X13824511650498

**Published:** 2014-01

**Authors:** D Carradice

**Affiliations:** Hull and East Yorkshire Hospitals NHS Trust,UK

**Keywords:** Venous insufficiency, Endothermal ablation, Sclerotherapy

## Abstract

This review presents the common diseases associated with superficial venous insufficiency of the leg. These include varicose veins, swelling, skin damage and ulceration. The benefits and rationale behind treatment are discussed, followed by the historical advances from ancient mortality and prayer to the modern endovenous revolution. Finally, an overview of modern treatment options will discuss the evidence supporting the gold standard of endothermal ablation and the cost effectiveness of treatment at this time of challenging resource limitation.

‘For I have entered into the fire and have come forth from the water...’ So begins the Ebers Papyrus, the oldest known record of the attempted treatment of superficial venous insufficiency. The reader is cautioned against the treatment of leg serpents, describing death presumably from haemorrhage or infection. Despite this inauspicious case series, humankind has continued to revisit this vexatious condition over the last 5,400 years. Recent advances in treatment have improved outcomes and are shown to be among the most cost effective surgical procedures available.^1,2^

## Definition, pathophysiology and prevalence

Superficial venous insufficiency (SVI) of the leg is defined as retrograde flow in the superficial veins of >0.5 seconds in duration. It is caused by a pathophysiological cycle initiated and driven by inflammatory processes that damage the vein walls and its valves. The superficial venous system is much more commonly affected but this can commence in any part of the venous tree and tends over time to propagate distally and proximally as well as between superficial and deep systems.

The earliest clinical features are usually varicose veins (from the Greek meaning ‘grape-like’), which are an observation of the most superficial segments of diseased vein. As venous hypertension increases, tissue changes become evident. The hydrostatic effects at the level of the capillary cause oedema, and further inflammatory processes result in skin and soft tissue damage such as haemosiderosis (pigmentation), venous eczema, lipodermatosclerosis (a fibrotic tightening of the soft tissues) and ulceration, which is often stubborn, tending towards chronic and relapsing disease.

Large population-based epidemiological studies have established that SVI is one of the most prevalent causes of disease facing Western healthcare systems. Between 30% and 50% of the adult population have uncomplicated varicose veins, and 3–10% have evidence of soft tissue damage with around 1% suffering venous ulcer disease.^3–7^

## Rationale for treatment

While it is rare for SVI to be a cause of death, it is associated with physical symptoms that decrease the quality of a sufferer’s life. In fact, this quality of life (QoL) impairment has implicitly or explicitly provided the drive to treat or attempt to treat this condition for thousands of years.

**Figure 1:**
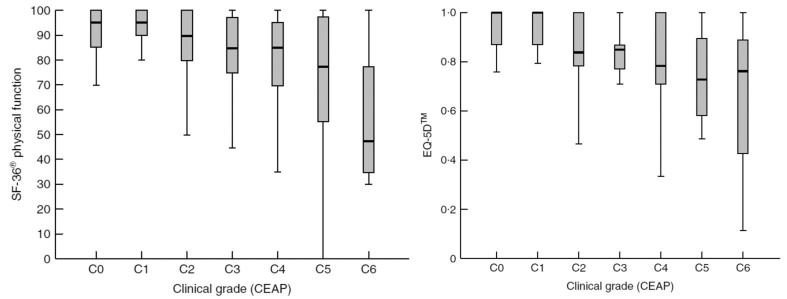
The association of venous insufficiency with physical function and index quality of life. Physical function is derived from the SF-36® questionnaire and is measured up to a maximum quality of life of 100%. Index quality of life is derived from the EQ-5D™ time trade-off estimation and measures quality of life as a proportion of full health (score = 1). The CEAP (Clinical, Etiological, Anatomical and Pathophysiological) system has six clinical categories (C0: no visible evidence of venous insufficiency; C1: thread veins; C2: varicose veins; C3: oedema; C4: skin changes including pigmentation, eczema and lipodermatosclerosis; C5: healed venous ulcer; C6: active venous ulcer).

**Figure 2:**
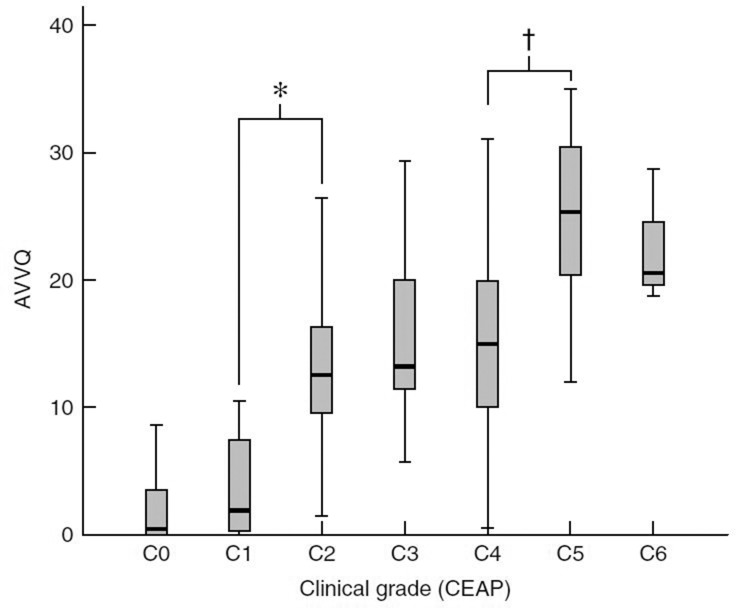
The association of venous insufficiency with impairment in quality of life. Quality of life is measured using the disease specific Aberdeen Varicose Vein Questionnaire (AVVQ) score (0 = no impairment). The CEAP (Clinical, Etiological, Anatomical and Pathophysiological) system has six clinical categories (C0: no visible evidence of venous insufficiency; C1: thread veins; C2: varicose veins; C3: oedema; C4: skin changes including pigmentation, eczema and lipodermatosclerosis; C5: healed venous ulcer; C6: active venous ulcer).

A detailed controlled observational study recorded QoL data for 456 patients with increasing severity of SVI and compared them with control data from 105 patients.^8^ The finding was that SVI was associated with a significant deterioration in quality adjusted life years (QALYs). This was associated specifically with loss of physical function, role limitation due to physical disability, bodily pain, general health and vitality (Fig 1). In those reporting physical symptoms, SVI had no impact on the domains associated directly with cosmetic concern such as mental health.^9^ This deterioration was evident even with uncomplicated varicose veins and, in fact, when using the most sensitive index of QoL impairment in venous dysfunction, there was no further deterioration moving from those without to those with soft tissue damage short of ulcer disease (Fig 2). This fact denies support for the practice of rationing treatment to only those who have developed skin changes.

There is a plethora of studies demonstrating significant improvements in QoL following successful treatment of SVI^2,10–15^ and this is of a similar magnitude to the improvement seen following a laparoscopic cholecystectomy or inguinal hernia repair.^16,17^ It is important to note that not all patients have symptoms; furthermore, physical symptoms such as aching and pain are common even in the absence of significant SVI. Patient selection is therefore important and the improvement of symptoms with compression can be used as a guide as to whether these are venous in origin.

The second established rationale for the treatment of SVI is in the management of venous ulcer disease. The ESCHAR (Effect of Surgery and Compression on Healing And Recurrence) trial randomised 500 patients with healed or active venous ulcers to receive either compression alone or compression and open surgery.^18^ The study was designed to detect a difference in ulcer recurrence rather than healing but did show that the addition of surgery was associated with lower recurrence at four years (31% vs 56% overall, improving to 27% vs 51% when only SVI was present and the deep veins were normal).

A recent Cochrane review aiming to evaluate the effect of superficial thermoablation on ulcer healing and recurrence found a small pseudorandomised study comparing compression with and without endothermal ablation of the insufficient superficial veins.^19^ Allocation of the first patient was random, with alternation used after this. This study suggested improved healing rates and less recurrence following ablation.^20^ This needs to be confirmed in a larger randomised study.

Finally, over time, SVI tends to be associated with a progression from uncomplicated disease to soft tissue damage and ulceration. In the absence of intervention, 27% of 116 diseased legs in 90 patients had progression of duplex findings over a median of 19 months and 11% had progression of clinical grade.^21^ In the Bonn vein study, the rate of progression in 1,978 patients was 31.8% over 6.6 years.^22^ There is weak evidence from a ‘before and after’ study that early diagnosis and corrective surgery for SVI can reduce the rate of venous ulceration on a population level.^23^

## Treatment

Now that a clear rationale of treatment is established, the next stage is to discuss which treatments should be offered to patients in need.

### Progress through the ages

The ancient Egyptian advice to avoid attempted treatment appears to have survived into ancient Greece, where effigies of legs with varicose veins were offered to the god Asclepius in the hope of relief. This process is depicted on a stone tablet in the National Archaeological Museum in Athens. Bandaging of ulcers was also inherited from Egypt and this was taken further in the fourth century BC when excess moisture was determined to be detrimental. The teachings of Hippocrates went on to recommend regular cleaning, debridement and removal of excess granulation. At the same time, further attempts were made to treat varicosities physically with incision, this time using cautery to reduce the risk of complications. Caution was recommended following the observation that incisions made into skin with the changes of chronic venous insufficiency may result in ulcer formation.

In the first century AD, the Roman physician Celsus advocated bandaging with compression for ulcers and described the successful excision of veins (phlebectomy). The general and seven-time consul Gaius Marius was said to have declined further treatment to his second leg, stating that ‘the cure was not worth the pain’, after experiencing phlebectomy without anaesthesia. The concept of segmental tributary phlebectomy was extended to treatment of the great saphenous vein (GSV) between the fourth and seventh centuries before ligation and stripping was described by Albucasis of Cordova (Al-Zahrawi) towards the end of the first millennium.

John Hunter’s work on thromboembolism in the 18th century included the first description of phlebitis but there was little progress seen in the management of SVI until ligation and stripping was developed and popularised at the end of the 19th century by Friedrich Trendelenburg, who noted five weeks of hospitalisation for recovery. He felt that his surgery was so fast that no attempt to dull the pain was required but it seems that patients did not agree and anaesthesia was first used for the procedure in Finland in 1897. Keller, Mayo and Babcock experimented with different stripping techniques 15 years later, and the point of ligation was moved up to the saphenofemoral junction itself, in the groin.

Following this, there was little change during the 20th century until its closing years when primitive experiments with electrocautery and diathermy led to the development of endothermal ablation. Treating SVI by injection had been described by Chassaignac in 1855 but sclerotherapy did not become popular until the middle of the 20th century, and this was relatively shortlived owing to high recurrence rates and complications. There was a renaissance of this approach with the development of foam sclerotherapy by the turn of the century.

### Modern management

Broadly speaking, modern physicians have four major categories of treatment available to them: conservative treatment, open surgery, endothermal ablation and endochemical ablation.

*Conservative treatment*: While conservative measures include the use of medication and lifestyle changes, the supportive evidence for these is relatively weak and the principal conservative measure in use is compression. This consists of hosiery or, in venous ulcer disease, compression dressings. Compression seeks to combat the relative venous hypertension and promote the antegrade flow of blood from the leg, improving calf muscle function and decreasing reflux. There is little doubt as to its benefits across the full range of disease, but uncertainty exists around the required degrees of compression and this is also an unpopular treatment with low compliance, even in venous ulcer disease where its role is undeniable.^24^ The reasons for this noncompliance are multifactorial and difficult to address.

Furthermore, if used for a prolonged period, compression treatment may be very expensive. The management of venous ulcer disease accounts for 2–3% of Western healthcare costs, with the compression dressings themselves making up the bulk of this. Compression hosiery is significantly cheaper but needs regular replacement and the costs mount over time.

*Open surgery – the one to beat:* This typically involves ligation of the major junctions (eg the saphenofemoral and saphenopopliteal junctions), communicating between deep and superficial venous systems, with or without the stripping of major superficial axial veins. A randomised trial involving 246 patients clearly demonstrated that it was significantly more effective than compression with sizable QoL benefits.^2^ In addition, it was shown to be highly cost effective across a ten-year time horizon. This is a conservative estimate as a large proportion of patients describe benefits for much longer following treatment.

Surgery is not without its drawbacks. In common with all invasive techniques, the procedure itself carries some morbidity, resulting in impaired QoL in the early postoperative period. Naturally, perioperative complications may exacerbate and extend this phenomenon. In good hands, conventional surgery on the whole is both safe and effective. Despite this, complications occur including infection (1.5–16%),^25–29^ haematoma (up to 33%),^30^ nerve injury (2–39%),^31,32^ deep vein thrombosis (up to 5%)^33^ and pulmonary embolism (0.2–0.5%).^28^ In the UK, the most common single cause of litigation following surgery is alleged injury to cutaneous nerves.^34^ These injuries are associated with pain, paraesthesia and anaesthesia. The rate of nerve injury is partially dependent on vein stripping, resulting in controversy surrounding below knee stripping of the GSV and stripping of the small saphenous vein (SSV). A failure to strip, however, reduces the efficacy and durability of the procedure.^35^

Reliable figures for long-term efficacy in terms of recurrence rates are difficult to obtain. Historically, few patients outside of trials have been followed up routinely, and saphenofemoral ligation and stripping was viewed previously as a good ‘trainee operation’, leading to some suggesting that technical failures by trainees is a possible explanation for the reported high recurrence rates. Furthermore, many argue that the now frequent practice of preprocedural duplex ultrasonography mapping^36^ has reduced the numbers of strategic rather than technical failures.

Nevertheless, even with this advantage, skilled surgeons (often with postoperative duplex imaging confirming the technical quality of the procedure) have reported that long-term results following conventional surgical treatment are marred by significant recurrence rates.^37^ Quoted rates are as high as 30% at one year, 40% at two years and up to 66% beyond ten years. Much of this recurrence is due to the growth of new incompetent vessels in old scar tissue (neovascularisation), significant unaddressed reflux in sections of vein that were not stripped for fear of nerve damage, untreated perforators and disease progression. Patients requesting reintervention for symptomatic recurrence are less common but, according to Hospital Episode Statistics data, approximately 15–20% of varicose vein procedures are performed for recurrent disease^38^ and the fear of recurrence has been shown to be a key concern for patients themselves, having a detrimental impact on satisfaction.

Innovations in open surgical technique have tried to address limitations and improve results. These include the use of barrier methods in the groin to reduce neovascularisation, locoregional anaesthesia, cryostripping and vein preserving surgery. These have been met with limited success and less enthusiasm, and there is little doubt that the pendulum is swinging away from open surgery in Western healthcare systems.

*Endothermal ablation – a new gold standard*: Endothermal ablation is the first of the endovenous techniques, which have made the requirement of general or regional anaesthesia obsolete and brought minimally invasive treatment into focus, with ‘walk in, walk out’ treatment (often in an office-based setting) a reality. Endothermal ablative techniques use heat to destroy the refluxing superficial veins, without the need to dissect and ligate them or to strip them out.

A treatment catheter or device is inserted percutaneously into the target axial vein (usually the GSV or SSV) under ultrasonography guidance. The catheter is inserted distally at the lowest point of incompetence and its tip positioned at or near to the junction. The vein is then surrounded with large volumes of very dilute local anaesthetic (tumescent). This results in pain relief but also performs several other important functions including compression of the vein on to the device (allowing the concentrated application of heat directly on to the venous endothelium) and hydrodissection (displacing surrounding structures such as the nerves and deep veins to safety). Finally, the tumescent acts as a heat sink (preventing thermal energy from propagating beyond the target vein). The use of tumescent anaesthesia therefore decreases morbidity and increases efficacy. The treatment device creates thermal energy at its tip and is withdrawn to treat the full length of the vein. This thermal energy obliterates the vein wall structure. The treated vein then heals, leaving behind only a cord-like structure of scar tissue and a tiny puncture mark on the skin where the device was inserted.

Thermal energy may be delivered in a range of ways. Endovenous laser ablation (EVLA) uses laser energy, radiofrequency ablation uses an electrical current and, finally, a device licensed in continental Europe emits bursts of steam. While radiofrequency ablation became available slightly earlier, slow withdrawal rates, relatively poor occlusion rates^37^ and complications pushed early enthusiasts towards EVLA, which rapidly became the most popular endovenous technique.

Early case series established that EVLA (with the skilled use of tumescent anaesthesia) is safe, highly efficacious (with vein occlusion rates approaching 100%) and very popular with patients. An area of disagreement evolved around how best to manage the visible varicose veins themselves, following axial ablation. During open surgery, phlebectomy is performed whereby small stab incisions are made over these veins, allowing them to be avulsed. A small randomised trial was designed to answer the question of whether this should be performed at the time of EVLA (concomitantly) or whether treatment should be delayed, allowing the veins to regress.^39^ Fifty patients were randomised to receive either concomitant or delayed phlebectomy. Two-thirds of patients in the delayed group ultimately went on to request subsequent treatment and did not see the same QoL improvements until a year following the initial procedure.

This remains a contentious issue despite this evidence, with some feeling that concomitant treatment represents ‘overtreatment’, and it is not without its disadvantages. It certainly slows the procedure down, increasing costs and reducing operative efficiency. Moreover, in a fee-for-service healthcare system, multiple ongoing ‘top-up’ treatments and a prolonged follow-up period are popular with doctors. Conversely, single treatment sessions are popular with patients^40^ and in an ‘episode-based’ health economy, multiple sequential treatments and prolongation of follow-up are costly.

The main question was obviously whether EVLA resulted in the same or even better outcomes as open surgery. This was answered in a 280 patient randomised trial.^10,41^ Both treatments were shown to be safe and equally effective with significant improvements in QoL. Furthermore, the EVLA group had significantly less pain and disability in the weeks following treatment, with reduced recovery times. In addition, clinical differences had emerged by one year. Despite technically adequate surgery, 20% had early evidence of clinical recurrence compared with just 4% in the EVLA group (similar to the expected background incidence of new venous disease). This appears to have been related in part to the avoidance of neovascularisation and residual below knee SVI. Endothermal ablation therefore has significant short and perhaps long-term benefits over open surgery.

Radiofrequency ablation systems have continued to evolve and early evidence for the ClosureFast™ (Covidien, Dublin, Ireland) device in particular is encouraging; studies emerging suggest similar early occlusion rates to EVLA,^42^ with equivalent safety and perhaps marginally less postoperative pain than early EVLA technology. EVLA is also evolving, with changes in laser wavelength and fibre tip suggested to reduce the pain following EVLA further.

*Endochemical ablation – work in progress:* Sclerotherapy involves the injection of agents (typically detergents) into the incompetent veins in order to induce phlebitis, followed by endoluminal fibrosis. While liquid sclerotherapy is not a new idea, the contemporary advent of injecting foam under ultrasonography guidance rather than liquid has resulted in a resurgence of its popularity. A key advantage is that the injection of tumescent anaesthesia is not needed, making the procedure itself as near painless as possible. This, however, comes at a cost: the occlusion rates in most series lag significantly behind those of endothermal ablation,^37^ leading to a requirement for multiple treatments. Additionally, complications have been a concern with matting and pigmentation in 18%, and neuroembolic complications in up to 0.9%^43,44^ (although the majority are transient, such as migraine and visual disturbance).^36,37^

Despite the steady move towards endothermal ablation, the mantle of ‘tumescentless’ treatment has not been abandoned. The current endothermal technologies are frustrated in attempts to avoid tumescence but newer endochemical techniques hope to improve the efficacy and possibly the safety of sclerotherapy with devices such as ClariVein® (Vascular Insights, Madison, CT, US) (using mechanical denudation alongside liquid sclerotherapy) and VenaSeal™ (Sapheon, Morrisville, NC, US), which uses cyanoacrylate glue to cause vein closure. The impact of these new technologies on the dominance of endothermal ablation is yet to be seen.

## What about the money?

An economic modelling study from 2010 considered conservative treatment, surgery, endothermal ablation and foam sclerotherapy for uncomplicated SVI.^1^ The finding was that EVLA had the highest probability of being cost effective to a third party payer, costing an estimated £2,467 per QALY over conventional treatment. (A conventional willingness-to-pay threshold of £20,000 per QALY is used in Western healthcare systems.) This did not even account for the additional savings associated with concomitant phlebectomy. Treatment of SVI is therefore even more cost effective in the advent of minimally invasive technology.

## Conclusions

The last decade has seen a revolution in the way we think about and treat SVI. Open surgical techniques, which have remained similar in principle for centuries, have been replaced with a new gold standard of endothermal ablation. Performed with local anaesthesia, patients can walk away immediately from treatment, with minimal pain and a rapid recovery. Treatment is associated with significant QoL benefits that are sustained by a return to a background incidence of further disease development. Endovenous treatments are cost effective and within the estimated willingness-to-pay threshold for Western healthcare systems.
